# LRO biogenesis and function: what can we learn from mast cells?

**DOI:** 10.3389/fcell.2025.1613677

**Published:** 2025-06-25

**Authors:** Juan Eduardo Montero-Hernández, Kerui Zhang, Ulrich Blank, Gaël Ménasché

**Affiliations:** ^1^ Université Paris Cité, Imagine Institute, Laboratory of Molecular basis of altered immune homeostasis, INSERM UMR1163, Paris, France; ^2^ Université Paris Cité, Centre de Recherche sur l’Inflammation, INSERM UMR1149, CNRS ERL8252, Faculté de Médecine site Bichat, Paris, France; ^3^ Laboratoire d’Excellence Inflamex, Paris, France

**Keywords:** mast cells, lysosome-related organelle (LRO), secretory granules, LRO transport, LRO fusion, pre-formed inflammatory mediators

## Abstract

Lysosome-related organelles (LROs) are specialized compartments with cell type-specific roles. In mast cells (MCs), which are tissue-localized hematopoietic effector cells, LROs refer to secretory lysosomes also known as secretory granules (SGs) containing numerous pre-formed inflammatory mediators including proteases, proteoglycans, lysosomal enzymes, histamine and serotonin. Their release during MC activation is responsible for allergic, inflammatory manifestations, the fight against parasitic agents or the neutralization of toxins. Here, we provide an overview of knowledge describing the mechanisms underlying the biogenesis, secretion and biological functions of LROs in MCs. Decoding molecular mechanisms involved in LRO biogenesis and biology of MCs will benefit i) to other immune or non-immune cell types containing LROs and ii) can be exploited to design novel therapeutic approaches for the treatment of allergic and chronic inflammatory diseases caused by MC activation.

## 1 Introduction

Lysosome-related organelles (LROs) are specialized compartments of the endo-lysosomal system that share several key features with canonical lysosomes, including an endosomal origin, acidic environment, lysosomal hydrolases and lysosome-specific membrane proteins (Delevoye et al., 2019). Found in both hematopoietic and non-hematopoietic cells, these organelles play an essential role in various physiological processes, such as pigmentation, bone remodeling, glucose regulation, lung plasticity, hemostasis and immune responses, by storing and controlling the secretion of specific contents (Banushi and Simpson, 2022). LRO secretion occurs in response to specific signals or stimuli which vary according to cell type.

This review focuses on LROs in mast cells (MCs). MCs are granulated cells of the hematopoietic lineage that reside in most tissues, particularly at epithelial and mucosal surfaces exposed to the external environment, including the skin, the airways, and the intestine (Beghdadi et al., 2011; Galli et al., 2020a). They are morphologically characterized by their high content of electron-dense LROs, commonly known as secretory granules (SGs). Due to their metachromatic staining with various cationic dyes, MCs were discovered in the late 19th century by the Germain scientist Paul Ehrlich, who referred to them as “Mastzellent” (meaning “well-fed” cells) because of their granule-filled appearance (Blank et al., 2013). MC LROs contain a variety of pre-formed inflammatory mediators, including proteases, proteoglycans, lysosomal enzymes such as β-hexosaminidase and vasoactive amines like histamine and serotonin (Wernersson and Pejler, 2014). Upon activation, MCs release these pre-formed mediators into the extracellular environment through a process known as MC degranulation (Menasche et al., 2021). Additionally, MCs also secrete newly synthesized lipid-derived mediators (such as leukotrienes and prostaglandins) produced from plasma membrane lipids and a variety of cytokines/chemokines, and growth factor via a distinct secretory pathway originating from the Golgi (Blank et al., 2014; Blank and Rivera, 2004). MCs are known as key effector cells in allergies resulting from an inappropriate immune response towards nonpathogenic products or allergens that lead to the production of allergen-specific IgE antibodies (Abs) (Galli et al., 2008; Charles and Blank, 2025). The aggregation of allergen-specific IgE bound to high-affinity IgE receptors (FcεRI) on MCs triggers a complex intracellular signaling cascade resulting in the release of LRO contents, lipid mediators and cytokines/chemokines (Menasche et al., 2021; Blank et al., 2021). These mediators rapidly initiate local tissue responses, including histamine’s well-characterized vasoactive effects and protease-mediated tissues permeability or leukotriene-mediated bronchoconstriction. In the long term, the release of chemokines and cytokines attracts other immune effector cells and contributes to immunoregulatory processes. Due to their location in tissues adjacent to nerve endings and blood vessels, and their expression of a wide variety of receptors (e.g., Toll-like receptors, complement receptors, neuropeptide and neurotransmitter receptors, lipid mediator receptors …), MCs can respond to a highly diverse array of stimuli (e.g., neuropeptides, complement fragments, cationic compounds, environmental substances etc.) (Redegeld et al., 2018). This versatility enables them to function as sentinel cells at the interface between innate and adaptive immunity, providing defense against parasites, bacteria, fungi and viruses. They also play a crucial role in venom detoxification and have more recently been implicated in nociception and behavioral changes, as evidences by food avoidance behaviors (Plum et al., 2023; Florsheim et al., 2023).

In this review, we will discuss recent advances in understanding the mechanisms underlying the biogenesis, secretion and biological functions of LROs in MCs.

## 2 Biogenesis and content of secretory granules

### 2.1 Biogenesis and maturation LRO

While much of our understanding of MC LRO biogenesis comes from studies on other cell types, such as cytotoxic T lymphocytes, neuroendocrine cells, and melanocytes, significant research has also been conducted directly on MCs (Wernersson and Pejler, 2014). In MCs, LRO biogenesis is initiated at the trans-Golgi network, where pro-granules form and undergo homotypic fusion to generate immature LROs (Hammel et al., 2010; Hammel et al., 1985). During maturation, granule contents progressively condense in a pH-dependent manner reducing organelle volume and achieving ultrastructural refinement (Hammel et al., 2010). These immature LROs continue to mature through sequential fusion events some occurring at the intersection of endocytic and exocytic pathways, forming hybrid organelles (Azouz et al., 2014a). This maturation phase is mediated by the small Rab GTPase Rab5, which promotes fusion between newly formed LROs and early endosomes, thereby regulating their size, composition and number (Azouz et al., 2014b). An intriguing observation in the RBL-2H3 mast cell line revealed that the depletion of synaptotagmin III - a protein involved in the regulation of the endocytic recycling compartment (ERC) - resulted in enlarged LROs (Grimberg et al., 2003). These findings suggest a potential functional cross-talk between the ERC and LROs, facilitating the removal and recycling of materials during LRO maturation. Such regulated maturation processes contribute to the morphological diversity of LROs, as evidenced by three distinct SG types observed under the electron microscope: type I SGs characterized by numerous intraluminal vesicles accessible to endocytic tracers, type II SGs presenting a dense core surrounded by intraluminal vesicles still accessible to endocytic tracers, and type III SGs containing only an electron-dense core that is no more accessible to endocytic tracers. Interestingly, type II SGs are proposed to arise from fusion events between types I and type III granules (Raposo et al., 1997). Furthermore, secretogranin III, a member of the granin family, appears to play a crucial role in MC granulogenesis through its interaction with chromogranin A (Prasad et al., 2008). Notably, overexpression of secretogranin III alone is sufficient to induce an expansion of the granular compartment (Prasad et al., 2008). New evidence also suggests cooperation between autophagy and endocytic pathways in LRO biogenesis, which depends on extracellular communication and facilitates the release of exosomes with preformed mediators (Omari et al., 2024).

Recent studies have also revealed that type I interferons (IFN-I) limit MC effector functions by suppressing LRO biogenesis (Kobayashi et al., 2019). Specifically, mouse Ifnar^−/−^ MCs exhibit enhanced LRO formation, characterized by enlarged organelle size and elevated content levels. Strikingly, this phenotype correlates with upregulated expression of TFEB - the master transcriptional regulator of lysosomal biogenesis - suggesting that IFN-I signaling may modulate LRO production through TFEB-dependent pathways. Further supporting the role of TFEB in MC LRO biogenesis, the same group demonstrated that the amino acid transporter SLC15A4, which acts downstream of IFNAR signaling, regulates TFEB function and LRO biogenesis (Kobayashi et al., 2017).

The acidic lumen of MC LROs is maintained by the vacuolar ATPase (V-ATPase) activity, a critical proton pump that facilitates the tight packing of granules constituents into the electro-dense core. Its pharmacological inhibition with bafilomycin A1 disrupts this pH gradient, resulting in significant alkalization of LROs (Pejler et al., 2017). This perturbation profoundly alters LRO morphology - causing granule swelling and vacuolization - and affects the storage of key mediators, including histamine, carboxypeptidase A3 (CPA3), and tryptase (Pejler et al., 2017).

**FIGURE 1:**
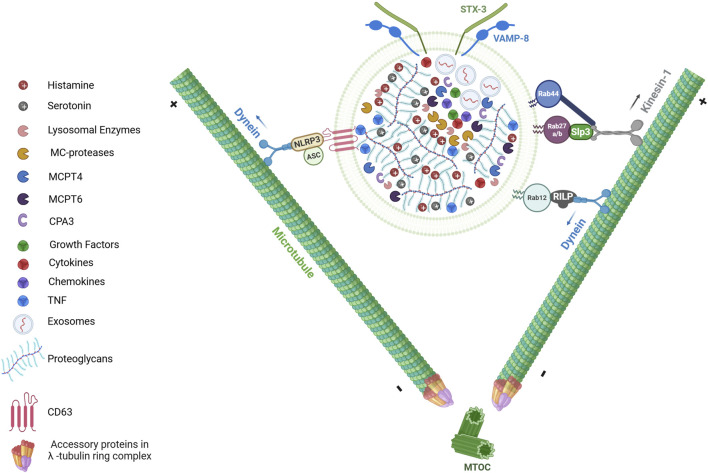
LRO composition and its transport upon activation. The secretory granules (SGs) of MCs, also referred to as lysosome-related organelles (LROs), serve as reservoirs for preformed inflammatory mediators. At ultrastructural level, LROs are heterogeneous and characterized by a dense core formed through electrostatic interactions between negatively charged heparin or chondroitin sulfate proteoglycans and positively charged insoluble mediators (e.g., TNF, MC-specific proteases (MCPT4, MCPT6, CPA3), histamine and serotonin). The granules also contain lysosomal enzymes (including β-Hexosaminidase and β-Glucuronidase), growth factors (such as TGFβ1, CXCL8, VEGF, SCF, PDGF FGF, NGF, FGF2), as well as cytokines and chemokines. Additionally, MC LROs can also contain exosomes, which are small extracellular vesicles (EVs) ranging from 30 to 80 nm in diameter. Upon activation, MCs release the contents of their LRO into the extracellular environment through a process known as MC degranulation. This process requires the bidirectional movement of LROs along the microtubule network. Anterograde transport (toward microtubule plus-ends) and retrograde transport (toward minus-ends) are both essential for proper LRO trafficking. Several proteins complexes involving members of Rab GTPases (such as Rab12, Rab27b, Rab44) regulate LRO movement by recruiting distinct microtubule-dependent motor proteins such as dynein and kinesin-1. In addition to Rab GTPases, inflammasome components such as NLRP3 and ASC have been implicated in SG trafficking through the recruitment of dynein. “Figure created with BioRender.com”.

The dense core of MC LROs plays a crucial role in their biogenesis and structural organization (Wernersson and Pejler, 2014; Moon et al., 2014). This core consists of proteoglycans covalently linked via glycosidic bonds to glycosaminoglycans (GAGs). In MCs, the most abundant proteoglycan is serglycin linked to heparin and chondroitin sulfate (GAGs) (Ronnberg and Pejler, 2012). These GAGs are highly sulfated and negatively charged facilitating electrostatic interactions with cationic mediators, such as histamine, tryptase, and chymase (Figure 1) (Ronnberg et al., 2012). Serglycin-deficient mice exhibit severely affected MCs, with granules losing their electron dense appearance (Abrink et al., 2004). The storage of several granules components is disrupted, including MC protease 4 (Mcpt4), Mcpt6, and carboxypeptidase A3 (CPA3). Mcpt2 chymase storage showed partial dependence on LRO, while Mcpt1 chymase and Mcpt7 tryptase (also known as TPSAB1) remain unaffected (Braga et al., 2007). Similarly, genetic deletion of NDST-2, an enzyme essential for heparan sulfate biosynthesis, drastically reduces the population of connective-tissue-type MCs. In contrast, mucosal MCs, which lack heparin, remain unaffected. NDST-2 deficiency diminishes protease storage in LROs, which exhibit sparse granules and empty vacuoles (Forsberg et al., 1999; Humphries et al., 1999). Likewise, the combined deficiency of several murine MCs proteases (i.e., mMcpt4, mMcpt5, mMcpt6, CPA3), as well as histamine in LROs results in abnormal granule morphology and less dense proteoglycan packing (Wernersson and Pejler, 2014; Nakazawa et al., 2014). These results highlight the bidirectional interdependence between proteoglycans and proteases and histamine in maintaining granule homeostasis (Grujic et al., 2013).

A distinctive feature of MCs is the prolonged period - often lasting several days or even months - between the formation of mature LROs and their eventual secretion (Hammel et al., 2010). This prolonged interval allows continuous maturation of the granules, during which they are also enriched with components from the extracellular environment. MCs actively endocytose these external elements, such as tumor necrosis factor (TNF), incorporating them into their granules (Figure 1) (Olszewski et al., 2007). This process of refining and acquiring additional bioactive molecules enhances the functional capacity of MC granules prior to their release.

### 2.2 LRO content and heterogeneity

MCs display an important heterogeneity including in their granular compartment depending on the species and the specific tissues in which they reside. Initially, MCs were classified into two main types based on histochemical staining and fixation methods, which are connective tissue MCs (CTMCs) and mucosal MCs (MMCs) in rodents (Enerback, 1966a; Enerback, 1966b). They also differ in proteoglycan content and granular proteases as for example, murine CTMCs express Mcpt6, and Mcpt7 tryptase, and Mcpt4 and Mcpt5 chymases, and the heparin proteoglycan while MMCs essentially contain the two chymases Mcpt1 and Mcpt2 and chondroitin sulfate (Pejler et al., 2007). In humans, MCs were categorized according to their expression of neutral proteases into MC_TC_ (containing tryptase and chymase), and M_T_ (containing tryptase only) (Irani et al., 1986). This traditional classification is evolving quickly with the development of single-cell transcriptomic analyses across various organs. For instance, in mice, CTMCs have been found to express the MC-specific G protein-coupled receptor MrgprB2, although expression levels and other key markers vary by tissue, while MMCs are MrgprB2-negative (Tauber et al., 2023). In humans, such analyses have uncovered a broader spectrum of MC diversity beyond the classical dichotomy, identifying up to six distinct transcriptionally defined MC clusters in nasal polyps and the intestine (Tauber et al., 2023; Dwyer et al., 2021).

Although partly heterogenous in nature, MC LROs contain a diverse array of preformed inflammatory mediators with critical roles in inflammatory responses, host defense, immunoregulation and tissue remodeling (Wernersson and Pejler, 2014). Estimates of their numbers in MCs are highly variable and range between 200 and 1500 depending on the author (Blank et al., 2014; Hamm et al., 1989; Krystel-Whittemore et al., 2015; Tanaka and Takakuwa, 2016).

The mediators contained in LROs can be categorized into several classes based on their biochemical nature and functional roles (Table 1). Proteoglycans such as heparin and chondroitin sulfate have important functions in mediator storage and anti-coagulant activity. Heparin can also modulate the activity, stability, and signaling of various growth factors through its electrostatic interactions (Koledova et al., 2019). Heparin expression begins early in fetal MCs and gradually increases over time. In mice, heparin is detectable as early as embryonic day 12.5 (E12.5) (Msallam et al., 2020). By embryonic day 17.5 (E17.5), fetal MCs express the neonatal Fc receptor, enabling sensitization by maternal IgE. At this developmental stage, these MCs are fully functional and capable of degranulation, underscoring their role in early immune responses (Msallam et al., 2020).

**TABLE 1:** Mediators contained in LROs and some of their associated biological function**s**.

Mediator Type	Mouse MCs	Human Mcs	Biological functions^a^ ^,^ ^b^
Biogenic Amines	Histamine, Serotonin	Histamine, Serotonin (low amounts)	Histamine: Vasodilation, increased vascular permeability (edema), itching, smooth muscle contraction, gastric acid secretion Serotonin: Smooth muscle contraction, neurotransmission modulation
Proteases	Chymases (Mcpt1, 2, 4, 5, 9) Tryptases (Mcpt6, 7) Carboxypeptidase A3 (CPA3)	Tryptase Chymase Carboxypeptidase A3 (CPA3)	Tryptase/Chymase: Tissue remodeling, pathogen defense (bacterial/toxin neutralization/degradation), neuropeptide processing, cytokine activation CPA3: Cleavage of C-terminal amino acids from peptides (e.g., endothelin-1), matrix degradation, toxin degradation
Lysosomal Enzymes	- β-Hexosaminidase - β-Glucuronidase - Arylsulfatase - Cathepsin D	- β-Hexosaminidase - β-Glucuronidase - Arylsulfatase	β-Hexosaminidase/β-Glucuronidase: Glycosaminoglycan degradation, bacterial cell wall breakdown Arylsulfatase: Sulfatide metabolism, anti-inflammatory regulation Cathepsin D (mouse): Protein degradation, antigen processing
Proteoglycans	Heparin (CTMC) chondroitin sulfate (MMC)	Heparin (MC_TC_) chondroitin sulfate (M_CT_)	Serglycin: Granule matrix stabilization, electrostatic packaging of proteases/biogenic amines, anticoagulant activity (heparin) Chondroitin sulfate: Matrix interaction, immune cell recruitment
Cytokines^c^	TNF, IL4, TGFβ1		TNF: key proinflammatory cytokine IL4: Th2-type immune responses TGFβ1: tissue remodeling, fibrosis
Chemokines^c^	CXCL8 (IL8)		CXCL8: neutrophil recruitment
Growth factors^c^	VEGF, PDGF, FGF2, NGF		VEGF: angiogenesis; PDGF: proliferation, migration, angiogenesis, wound healing NGF: neuronal survival, differentiation, FGF2: tissue repair, angiogenesis, proliferation and differentiation

^a^ additional information can be found in reference (Wernersson and Pejler, 2014).

^b^ additional information can be found in reference (Pejler et al., 2007).

^c^ as indicated in reference (Mukai et al., 2018) note that presence in mouse or human mast cells was not specified.

An important MC mediator with many functions is histamine, a biogenic amine, which is synthesized from the amino acid histidine by histidine decarboxylase. It induces vascular permeability, vasodilatation, smooth muscle contraction and mucus secretion by binding to H1 receptors (Wernersson and Pejler, 2014; Simons, 2003). Some subsets of MCs also contain serotonin contributing to vascular permeability and vasodilation and many other functions including modulation of neural activities (Wernersson and Pejler, 2014; Breil et al., 1997). MC granules also contain a number of MC-specific proteases such as tryptases, chymases, and CPA3 (Pejler et al., 2007). Tryptases and chymases are serine protease, while CPA3 is a zinc-dependent metalloprotease (Pejler et al., 2007). As mentioned above these proteases vary depending on the MC type and species. They have numerous functions including in venom detoxification, tissue repair fibrosis etc that have been extensively reviewed previously (Wernersson and Pejler, 2014; Pejler et al., 2007; Caughey, 2016). In addition to these MC-specific proteases, MCs also secrete proteases shared with other cells that result at least in part from their endo-lysosomal nature. They include matrix metalloproteases 9 (MMP9) (Baram et al., 2001), cathepsin A, B, C, D, and E (Wernersson and Pejler, 2014; Pejler et al., 2007; Caughey, 2016; Dragonetti et al., 2000; Wolters et al., 2000; Henningsson et al., 2005) and a variety of others such as angiotensin II generating renin important for blood pressure regulation (Silver et al., 2004) or proapoptotic granzyme B (Pardo et al., 2007). LROs also contain lysosomal enzymes including β-Hexosaminidase and β-Glucuronidase. They are widely used as a biochemical marker to assess MC degranulation. These enzymes degrade glycosaminoglycans, such as heparan sulfate and chondroitin sulfate or hyaluronan, facilitating tissue remodeling during inflammation and immune responses (Gushulak et al., 2012; Griffin and Gloster, 2017). β-Hexosaminidase also degrades bacterial peptidoglycan, a critical component of bacterial cell walls (Fukuishi et al., 2014). *In vivo*, mice lacking β-hexosaminidase show increased lethality of bacterial infections, confirming its role in host defense (Fukuishi et al., 2014). Although MCs are known to secrete a large variety of newly synthesized cytokines, chemokines and growth factors (Mukai et al., 2018), a distinctive feature of MCs is that they are able to secrete some of them from a preformed pool stored in their LROs making them available immediately without the requirement of new synthesis. Thus, in addition to TNF described early on (Gordon and Galli, 1990), several of them have been reported to get released from prestored sources including TGFβ1, CXCL8, VEGF, SCF, PDGF FGF, NGF, FGF2 (Mukai et al., 2018). Concerning TNF it was shown that after transient exposure in its membrane-expressed form at the cell surface, it can get reinternalized before being stored as a preformed mediator (Olszewski et al., 2007). Whether reinternalization of basically released cytokines/chemokines/growth factors represents a general mechanism valuable for their storage in preformed form remains an open question.

Mast cell LROs also contain exosomes, which are small extracellular vesicles (EVs), typically 30–80 nm in diameter (Figure 1). They originate from the endosomal system through maturation of early endosomes into late endosomes, in which intraluminal vesicles (multivesicular bodies, MVBs) are generated through the inward budding of the endosomal membrane. Upon release, exosomes play a crucial role in cell-cell communication by transferring bioactive molecules such as proteins, lipids, and nucleic acids (mRNAs, miRNAs, and lncRNAs) to a recipient cell thereby regulating immune responses and other biological processes (Elieh-Ali-Komi et al., 2025).

## 3 LRO transport and secretion

### 3.1 LRO transport

LROs in MCs exhibit bidirectional movement along the microtubule network, involving both anterograde transport (toward microtubule plus-ends) and retrograde transport (toward minus-ends) (Figure 1) (Brochetta et al., 2014; Nishida et al., 2005; Smith et al., 2003). The anterograde transport of LROs to the plasma membrane is mediated by kinesin-1, an archetypal member of the kinesin superfamily (Munoz et al., 2016). Kinesin-1 comprises two heavy chains (KIF5A, KIF5B, or KIF5C) and two light chains (KLC1, KLC2, KLC3, or KLC4), with KIF5B and KLC1 predominantly expressed in MCs. A conditional murine model lacking *Kif5b* specifically in hematopoietic cells, including MCs, demonstrated that kinesin-1 regulates LRO transport to exocytosis sites during FcεRI activation (Figure 1) (Munoz et al., 2016). Upon stimulation, kinesin-1 interacts with the Slp3/Rab27b complex on LROs through the Slp homology domain (SHD) of Slp3. The assembly of this kinesin-1/Slp3/Rab27b trimeric complex depends on the phosphatidylinositol 3-kinase (PI3K) activity, which regulates kinesin-1 accessibility to Slp3 as a cargo receptor (Munoz et al., 2016). Retrograde transport, on the other hand, is mediated by dynein, which directs the movement of LROs towards the ends of microtubules in the perinuclear region, thus counterbalancing anterograde trafficking (Efergan et al., 2016). More recently, it was shown that the inflammasome components NLRP3 and ASC play a critical role on MC degranulation by orchestrating LROs trafficking (Figure 1) (Mencarelli et al., 2024). Following IgE-Ag activation, NLRP3 and ASC interact with the LRO membrane glycoprotein CD63, forming an inflammasome complex termed the “granulosome”. This complex recruits the motor protein dynein through an interaction with NLRP3, facilitating bidirectional microtubule-dependent transport of LROs to the plasma membrane. Interestingly, under lipopolysaccharide (LPS) stimulation, MCs secrete pro-IL1β via LROs, which is then converted extracellularly into active IL-1β by MC-derived proteases (e.g., chymase). This mechanism amplifies anaphylactic responses by coupling inflammasome signaling with protease-dependent cytokine maturation (Mencarelli et al., 2024).

Rab GTPase, small proteins of 20–25 kD, are crucial regulators of vesicular trafficking in both endocytic and exocytic pathways across all cell types. These proteins function as molecular switches, alternating between an active (GTP-bound) and an inactive (GDP-bound) forms (Stenmark, 2009). Over 60 Rab family members have been identified in the human genome, each localizing to specific membrane compartments to confer organelle identity and recruit trafficking machinery (Stenmark, 2009). Rab GTPases undergo post-translational prenylation at their C-terminal cysteine residues, enabling reversible membrane binding. A functional screening assay of 44 Rab proteins identified 30 potential regulators of MC LRO trafficking and exocytosis (Azouz et al., 2012). Among these, Rab27a and Rab27b are both expressed in bone marrow-derived MCs (BMMCs) and localize to LROs. Studies using single and double knockout mice for Rab27a and Rab27b demonstrated that the Rab27 family, particularly Rab27b, plays a crucial role in MC degranulation (Mizuno et al., 2007). Interestingly, Rab27a and Rab27b have distinct and sometimes opposing roles. Rab27a acts as a negative regulator through its action on actin, while both Rab27a and Rab27b act as positive regulators through their interaction with Munc13-4 (Singh et al., 2013). Another negative regulator of MC degranulation is Rab12, which mediates microtubule-dependent retrograde transport of LROs. Upon MC activation, Rab12 interacts with the Rab-interacting lysosomal protein (RILP) within the RILP-dynein complex to transport LROs towards the microtubule minus-end in the perinuclear region (Figure 1) (Efergan et al., 2016). The Rab GTPase family has been recently expanded to include several large Rab GTPase members, such as CRACR2A, (Rab46), Rab45 and Rab44 (Srikanth et al., 2017). These proteins share a conserved C-terminal Rab domain, which is linked to additional functional domains, including an EF-hand domain, a coiled-coil domain, and a proline-rich domain (Srikanth et al., 2017; Tsukuba et al., 2021). Interestingly, in MCs only Rab44 is expressed. Rab44, has been implicated in MC degranulation and IgE-mediated anaphylaxis, (Kadowaki et al., 2020; Longe et al., 2022). It interacts with kinesin-1 to regulate LRO translocation to the plasma membrane upon FcεRI activation (Figure 1) (Longe et al., 2022). This process relies on the recruitment of Rab44 to LROs through its Rab GTPase domain and operates independently of Ca2+ signaling (Longe et al., 2022).

Munc18-2, an isoform of the mammalian uncoordinated18 (Munc18) protein family, plays multifaceted roles in MC LRO dynamics. In addition to its established function as a fusion accessory protein, Munc18-2 contributes significantly to LRO translocation (Brochetta et al., 2014). The association of Munc18-2 with LROs is dependent on microtubule integrity. Indeed, microtubule destabilization with nocodazole redistributed Munc18-2 from granular structures to a diffuse cytosolic pattern, demonstrating that its recruitment to LROs depends on an intact microtubule network. Upon cellular stimulation, Munc18-2 translocates along microtubules to the cell periphery, where it associates with fused LROs in forming lamellipodia. During this process, the interaction between Munc18-2 and β-tubulin diminishes, suggesting a dynamic regulation with the microtubule cytoskeleton (Brochetta et al., 2014). In Munc18-2-depleted cells, LROs appear stationary, remaining docked along intracellular microtubules (Brochetta et al., 2014). This observation suggests that Munc18-2 may dynamically dock LROs during microtubule-based transport, potentially in concert with the kinesin-1/Slp3/Rab27b/Rab44 transport mechanism. Supporting this hypothesis, neuronal Munc18-1 binds the kinesin-1 adaptor FEZ1 to mediate axonal vesicle transport, highlighting an evolutionarily conserved link between Munc18 proteins and microtubule-dependent trafficking (Chua et al., 2012).

### 3.2 LRO fusion machinery regulating MC secretion

**FIGURE 2:**
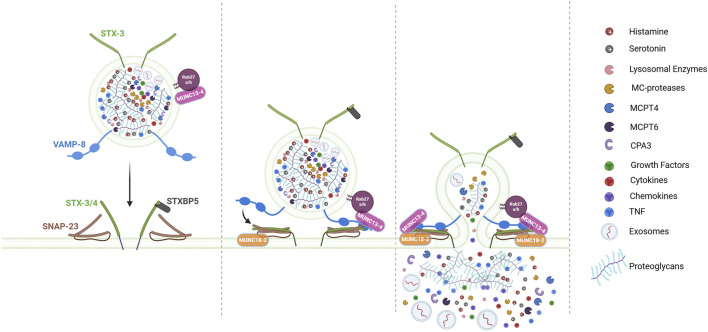
Model for LRO docking/fusion machinery during MC degranulation. The fusion of LROs between themselves (not shown) and the plasma membrane (PM) is not a spontaneous event but is triggered by activation signals and controlled by a sophisticated membrane fusion machinery involving SNARE proteins (Soluble NSF Attachment Protein Receptor proteins) and their regulatory proteins. In MC, key SNARE components include the t-SNARE SNAP-23, STX3 and STX4, as well as the v-SNAREs VAMP8. Fusion is initiated by the assembly of a tetrameric trans-SNARE complex, which bridges the LROs and PM. This assembly is tightly regulated by accessory proteins such as MUNC13-4, MUNC18-2, STXBP5, which facilitate LRO docking at the PM and subsequent fusion. Following degranulation, MCs release the dense proteoglycan matrix of LROs, which contains electrostatically bound mediators (e.g., histamine, serotonin, TNF, MC-specific proteases), capable of long-distance transport to lymph nodes via the bloodstream. Simultaneously, soluble mediators (e.g., cytokines, chemokines), exosomes (30–80 nm extracellular vesicles), and growth factors diffuse into the extracellular space, also driving inflammatory and immune responses. “Figure created with BioRender.com”.

The discharge of LRO content by MC degranulation can occur following stimulation via various cell surface receptors. A potent stimulus is the aggregation of FcεRI-bound IgE by a multivalent antigen or allergen (Blank et al., 2021). This process can trigger the massive release of up to 100% of MC granule contents through a multigranular (compound) mode of exocytosis, which involves granule-granule and granule-plasma membrane fusion events (Menasche et al., 2021). Stimulation of other surface receptors, such as MRGPRX2, C3aR, C5aR, or ET1R, leads to a more selective release of individual secretory granules (SGs) located just beneath the plasma membrane (Gaudenzio et al., 2016). Under certain conditions, MCs can also undergo piecemeal exocytosis—a regulated secretory process in which SGs release their contents gradually and partially rather than undergoing full fusion with the plasma membrane. This allows for dynamic modulation of secretion by selectively releasing specific mediators while retaining others (Dvorak et al., 1994; Dvorak, 2005). Once released, positively charged mediators such as histamine and proteases diffuse away due to associated pH changes (Wernersson and Pejler, 2014; Galli et al., 2005). It is important to note that, contrary to some misconceptions in the literature, MC degranulation does not involve the release of entire granules but rather the expulsion of their densely packed proteoglycan matrix contents, which then disperse into the surrounding tissue (Figure 2).

The fusion of LROs between themselves and the plasma membrane is not a spontaneous event but a process controlled by a sophisticated membrane fusion machinery involving SNARE (Soluble NSF Attachment Protein Receptor proteins) and SNARE accessory proteins (Figure 2) (Menasche et al., 2021; Hong, 2005). SNARE proteins contain a 60–70 amino acid motif with heptad repeats that form coiled-coil structures. This motif allows them to assemble into tight, four-helix bundles called trans-SNARE complexes the formation of which is energetically favored (Sutton et al., 1998). This drives fusion bringing opposing membranes into close proximity, allowing lipid bilayer merging (Hong, 2005; Jahn and Scheller, 2006). SNARE proteins can be divided functionally into vesicular SNAREs and target SNAREs, the former being composed of the family of vesicular associated membrane proteins (VAMPs), the latter containing the family of Syntaxin (STX) proteins and the Synaptosomal associated protein (SNAP23/25) family. As intracellularly fusion events occur also between vesicles only they have also been classified according to structural principles into R-SNAREs containing a central R residue and Q-SNAREs containing a central Q residue. The Q-SNAREs can be further divided into Qa, Qb and Qc SNAREs (Jahn and Scheller, 2006; Kloepper et al., 2007). STXs represent Qa SNAREs while SNAP23/25 contain two helices of Qb and Qc SNAREs. Note, however that individual SNAREs containing either Qb and Qc SNAREs also exist (Kloepper et al., 2007). The final fusion-competent SNARE complex will then contain 1 R and the three Qa, Qb and Qc SNAREs.

The first SNARE proteins identified in MC degranulation was the Qb/Qc SNARE SNAP23 (Guo et al., 1998). It was localized at the plasma membrane in resting MCs, but upon stimulation relocates to the interior in agreement with its participation in compound exocytosis which involves granule-granule fusion events (Guo et al., 1998). Its functional implication was confirmed using Ab blocking experiments or studies with mutant SNAP-23 unable to associate with membranes (Guo et al., 1998; Agarwal et al., 2019). In SNAP-23 conditional KO mice MCs its direct implication could not be evaluated as these mice had a severe defect in MC development likely through its importance also in constitutive secretion events (Cardenas et al., 2021). For the Qa SNAREs both STX3 (localized on LROs and the plasma membrane) and STX4 (localized at the plasma membrane) isoforms have been implicated using knockdown experiments (Brochetta et al., 2014; Woska and Gillespie, 2011). However, further investigation in knockout cells and animals revealed a more complex picture as STX4 KO cells had no defect while STX3 KO cells showed a partial defect. No defect in whole animal anaphylaxis experiments were seen in STX3 KO mice supporting a role of additional STXs and/or compensatory effects (Sanchez et al., 2019).

Regarding the implication of R-SNAREs, VAMP8 appeared to represent a key R-SNARE in MC degranulation. Initially called endobrevin, because of its localization on endocytic vesicles, it colocalizes in MCs with LROs again revealing their endo-lysosomal nature (Tiwari et al., 2008). VAMP8-deficient BMMC released less histamine and β-hexosaminidase, while neosynthesized cytokine secretion remained intact. VAMP8-deficient mice also exhibit reduced passive anaphylactic responses (Tiwari et al., 2008). In addition, an enhanced and transient association of VAMP8 with SNARE partners STX4 and SNAP23 could be demonstrated in coimmunoprecipitation experiments during IgE-mediated stimulation (Tiwari et al., 2008).

Several other v-SNAREs, including VAMP7, VAMP2, and VAMP3, have also been investigated for their roles in MC degranulation. Knockdown of VAMP7 or its inhibition by antibodies reduces secretion in the RBL-2H3 MC line and human primary MCs (Woska and Gillespie, 2011; Sander et al., 2008). Upon stimulation, VAMP7 translocates to the plasma membrane, where it forms complexes with SNAP-23 and STX4 (Sander et al., 2008). However, its co-localization with LROs remains unclear, and VAMP7 knockout (KO) cells have not yet been studied. In RBL-2H3 MCs, fluorescently tagged VAMP2 translocated to the plasma membrane upon stimulation (Miesenbock et al., 1998). However, VAMP2-KO BMMCs exhibit no degranulation defect, indicating that VAMP2 is not essential for this process (Puri and Roche, 2008). Interestingly, in VAMP8-deficient MCs, VAMP2 showed an increased tendency to associate with SNAP-23 upon stimulation, suggesting a potential compensatory role (Tiwari et al., 2008). Similarly, VAMP3-deficient MCs do not display degranulation defects (Puri and Roche, 2008). However, in a VAMP8-deficient background, VAMP3 was found to interact with SNAP-23, though its role appeared to be limited to constitutive fusion rather than stimulated degranulation (Tiwari et al., 2009).

Although SNARE complexes can form spontaneously it is clear that in living cells this process is regulated by a variety of other proteins regulating their assembly, modulating energy barriers, and ensuring precise timing (Menasche et al., 2021; Blank et al., 2014; Jahn and Scheller, 2006). Sec1/Munc18-like (SM) proteins are a conserved family that interact specifically with certain STXs that play important roles in SNARE assembly (Sudhof and Rothman, 2009). Munc13 family proteins function in membrane vesicle docking interacting with Rab27-docked vesicles (Brunger et al., 2019; Rizo, 2022). It also primes fusion by converting STXs from a fusion-incompetent Munc18-bound closed conformation to an open conformation, making them available for SNARE complex formation thereby cooperating with Munc18 to finally enable SNARE assembly (Brunger et al., 2019; Rizo, 2022; Rothman et al., 2023). Studies with Munc18-2 and Munc13-4 isoforms knockout MCs and anaphylaxis experiments have found a profound degranulation defect in the absence of these proteins confirming their importance in the fusion process (Gutierrez et al., 2018; Rodarte et al., 2018). It is possible that Munc18-2, contrary to neuronal form Munc18-1, but like Munc18-3 (Hu et al., 2007), may bind to its STX partners (STX2 and STX3) already in an open form and thus may not need priming. Thus, both Munc18-2 and Munc13-4 may directly cooperate for proper assembly of SNARE fusion complexes. In this process Munc13-4 likely gets targeted to the granule fusion site via its interaction with Rab 27 prior to its activation by calcium signaling upon calcium binding to its C2A and C2B domains. As mentioned above granule-associated Munc18-2 (via STX3 binding) may additionally play a role in LRO docking and microtubule-dependent transport as after specific knock down LROs appear less docked at the plasma membrane and less mobile (Brochetta et al., 2014). Another calcium sensor in MCs is Synaptotagmin-2 (Syt2). Absence of Syt2 markedly inhibited degranulation in IgE-stimulated MCs and knockout animals showed a reduced passive cutaneous anaphylaxis response. The mechanism of action is not entirely clear. In neuronal cells Syt I is proposed to interact and clamp the preassembled SNARE prefusion complex diffusing away upon arrival of calcium, binding to its C2A and B domains thereby unlocking the prefusion complex to allow SNARE zippering. Another reported fusion regulator is tomosyn or STXBP5 initially described in neuronal cells, where its absence enhances neurotransmission (Fujita et al., 1998). Tomosyn possesses a R-SNARE domain but no membrane anchor and can bind to both STX 3 and 4 in MCs (Figure 2) (Madera-Salcedo et al., 2018). Here also, tomosyn acts as a fusion clamp as after siRNA-mediated knock down MCs exhibited an enhanced degranulation response (Madera-Salcedo et al., 2018) confirming the data in neuronal cells (Fujita et al., 1998), pancreatic β cells (Zhang et al., 2006) and endothelial cells (Li et al., 2018). In agreement with the fusion clamp function in MCs tomosyn rapidly dissociated from STX4 in a manner regulated by phosphorylation through PKCδ (Figure 2). By contrast the interaction with STX3 increased after stimulation for reasons that are not entirely clear (Figure 2). But it could represent a feedback regulatory mechanism to avoid uncontrolled fusion (Madera-Salcedo et al., 2018).

Together, these data demonstrate that, in addition to *bona fide* SNARE proteins, numerous additional regulators contribute to the fusion, docking, and trafficking processes, exerting both positive and negative regulatory roles. Among these, several Rab isoforms also play crucial roles, including Rab3d (Pomb et al., 2001), Rab5 (Klein et al., 2017), Rab12 (Efergan et al., 2016), Rab27a/b (Mizuno et al., 2007; Elstak et al., 2011), Rab37 (Higashio et al., 2016), and Rab44 (Longe et al., 2022). Other key regulators include the Rab27-interacting synaptotagmin-like protein 3 (Slp3) (Munoz et al., 2016), complexin II (Tadokoro et al., 2005), and Doc2α (Higashio et al., 2008), each playing distinct roles in fusion, granule docking, and trafficking, as summarized in previous reviews (Menasche et al., 2021; Blank et al., 2014; Moon et al., 2014). Furthermore, these processes require exquisite coordination with cytoskeletal reorganization, ensuring efficient transport mechanisms and enabling LROs and fusion effectors to access their respective membrane sites (Menasche et al., 2021).

## 4 The function of the LRO contents beyond its secretion

### 4.1 Role of LRO exocytosis on allergic manifestations

MCs and their mediators have been studied over many years primarily for their role in allergic manifestations. They are key effectors of IgE-mediated type I immediate hypersensitivity reactions, a Th2-mediated immune response involved in allergic responses. Allergic diseases affect nearly one-third of the population in developed countries, with a notable rise since the latter half of the 20th century. This increase is believed to result from an inappropriate immune response against nonpathogenic substances called allergens, such as pollen, dust mites, pet dander, mold spores etc. This response is favored - according to the hygiene hypothesis - in environments with higher hygiene standards, which reduce microbial exposure, disrupt the microbiota, and compromise barrier surfaces like those of the skin, lungs, and gut.

Allergies can present in various forms including anaphylaxis, allergic rhinitis, conjunctivitis, asthma, atopic dermatitis and food allergies. Anaphylaxis is the most severe form of IgE-mediated type I hypersensitivity, marked by rapid onset and potentially fatal systemic effects, while the other manifestations usually present as chronic diseases although in the case of asthma and food allergies they can present/evolve into severe forms with fatal outcome. MC-released histamine from LROs is a central mediator in the pathophysiology of allergies. Histamine is synthesized from the amino acid histidine by histidine decarboxylase (HDC) and acts on histamine receptors (in particular H1) in the body, triggering a range of responses (Wernersson and Pejler, 2014; Ohtsu, 2012). These include i) vasodilation and vascular permeability leading to a drop in blood pressure (hypotension) and tissue swelling (angioedema), ii) smooth muscle contraction inducing bronchoconstriction, contributing to respiratory symptoms like wheezing, shortness of breath, and airway obstruction in asthmatics iii) cardiovascular effects by increasing heart rate and cardiac contraction while reducing peripheral vascular resistance, potentially worsening circulatory collapse in anaphylactic shock iv) mucus secretion in the airways and gastrointestinal tract, which may result in congestion, rhinorrhea, conjunctivitis, sneezing vomiting, or diarrhea in allergic rhinitis and food allergies (Wernersson and Pejler, 2014; Galli et al., 2008). The importance of histamine in allergies is underlined by the significant impairment in the development of allergic reactions in HDC-KO mice (Ohtsu, 2012). Until today, although some more costly alternatives biologics targeting the IgE receptor or Th2 immune response development in severe diseases exist, anti-histamines represent the first line treatment for most chronic allergic manifestations. In addition to histamine other SG components can also participate in the early phases of allergies. Initially thought to be present in rodent MCs the biogenic amine serotonin has also been found in human MCs although in lower quantities (Wernersson and Pejler, 2014; Kushnir-Sukhov et al., 2007). Like histamine it contributes to swelling and redness by promoting vasodilation and increasing vascular leakage. It can potentiate histamine’s effects, worsening symptoms like angioedema and hypotension in anaphylaxis (Gillis et al., 2017). During an allergic reaction the MC-specific proteases chymase and tryptase contribute to leukocyte extravasation by modulating adhesion molecules on endothelial cells (Caughey, 2007). Likewise, MC proteases by degrading extracellular matrix proteins further facilitate inflammatory cell infiltration (Pejler et al., 2007). Tryptase activates protease-activated receptor-2 (PAR-2), which can induce airway smooth muscle contraction and increase mucus secretion can contribute to asthma symptoms (Caughey, 2007). A recent study has uncovered important insights into the pathophysiology of allergic asthma during early life. The findings showed that allergen-induced tissue remodeling disrupts the vascular integrity of the lung through MC degranulation, and more specifically through the release of proteases in mice and tryptase in humans. These proteases induce the retraction of pericytes, which are key cells for maintaining blood vessel stability, thus establishing a new MC/pericyte axis critical for lung vascular function (Joulia et al., 2024).

Following release of LRO content by degranulation, MCs also rapidly (within 15–30 min) release newly synthesized lipid mediators like prostaglandins (PGD2) and leukotrienes (LTB4, LTC4), triggering classic allergic symptoms such as vasodilation, increased vascular permeability, bronchoconstriction, and mucus production thereby amplifying allergic symptoms. These early phases are followed by a late-phase response, where MCs synthesize secrete chemokines, cytokines, and growth factors, attracting additional inflammatory cells like neutrophils, macrophages, eosinophils, and T cells to the inflammatory site that finally contribute to the chronicity of the allergic reaction.

A recent study by Lämmermann’s group reveals that during anaphylactic conditions, degranulating MCs can trap neutrophils intracellularly, forming structures referred to as “MC intracellular trap” (MIT) (Mihlan et al., 2024). This process relies on MC-dependent secretion of LTB4, MC degranulation, and neutrophil migration. Once recruited, MCs engulf live neutrophils into giant vacuoles, where the neutrophils undergo cell death within 48 h. The MCs then degrade and recycle neutrophil components (e.g., DNA, proteases), enhancing their metabolic fitness. Subsequently, the residual neutrophil-derived material is release by degranulation (known as “nexocytosis”), which amplifies inflammation by triggering type I interferon responses (Mihlan et al., 2024).

### 4.2 Role of LRO contents on venom detoxification

Despite their potentially life-threatening effects, IgE-mediated allergic responses have been preserved throughout evolution, likely due to their beneficial roles in Th2-mediated innate and adaptive immune responses (Palm et al., 2012; Charles and Blank, 2025). Indeed, M. Profet’s “toxin hypothesis” suggests that allergies function as a defense against harmful substances produced by organisms across the kingdom of living organisms (Profet, 1991). These include venoms and environmental toxins, but eventually also endogenously produced products that need to be neutralized. Supporting this, MC-derived products have been shown to neutralize toxins, venoms (snake, scorpion, gila monster, bee), plant toxins (poison ivy urushiol), and endogenous compounds like Endothelin-1 and vasoactive intestinal peptide (Galli et al., 2020b).

Major LRO components released by MCs in this task are the MC-specific proteases and proteoglycans neutralizing toxins and venoms through degradation (proteases) or binding (heparin) (Metz et al., 2006; Tsai et al., 2015; Du et al., 2024). Thus, in mice, CPA3 and the MC-specific chymase MCPT4 were demonstrated to degrade various venoms, while in humans, it appears to be majorly β-tryptase, which breaks down certain snake venoms (Tsai et al., 2015; Anderson et al., 2018). Heparin, can reduce venom toxicity by binding and blocking activity or by acting as an anticoagulant (Du et al., 2024).

Many studies suggest an innate MC-triggering mechanism in venom detoxification. Indeed, venom components like mastoparan (bee venom) and sarafotoxin (snake venom) activate the MRGPRX2 receptor on MCs, inducing degranulation. MCs also express endothelin A (ETA) receptors that get directly activated by endogenous endothelin-1 (ET-1) or exogenous sarafotoxin 6b contained in snake venoms (Tsai et al., 2015; Schneider et al., 2007). However, repeated venom exposure can also elicit an adaptive immune response, as demonstrated for bee venom phospholipase A2 (PLA2), which triggers a Th2-response and production of venom-specific IgE (Starkl et al., 2022). Thus, adaptive immunity, IgE, and FcεRI, despite its potentially dangerous effect can also further enhance protection against toxins and venoms.

### 4.3 Role of LRO exocytosis against microbial defense

MCs and IgE antibodies have been proposed for many years to contribute to parasitic defense mechanisms, in particular large parasites such as helminths with parasite-specific IgE increasing in endemic areas and correlating with protection (Hagan et al., 1991; Rihet et al., 1991). In 1996, MCs were also shown to play a protective role in bacterial sepsis (Echtenacher et al., 1996; Malaviya et al., 1996), followed by studies revealing both protective and aggravating roles of MCs in bacterial infection, depending on infection severity, entry site, and model (Abraham and St John, 2010; Jimenez et al., 2021). MCs also contribute to antiviral and antifungal defenses with both positive and negative roles (Rathore and St, 2020; Jiao et al., 2019).

While IgE-mediated adaptive mechanisms represent an important component during parasitic infection, MCs express also numerous innate receptors such as TLR, NOD-like and RIG-I-like receptor families, C-type lectin receptors and Mas-related G protein-coupled receptors that can recognize and respond to microbes or microbial products initiating the release of secretory products involved in defense mechanisms (Redegeld et al., 2018).

Concerning helminth infection, studies in mice confirmed the role of MCs and IgE-mediated adaptive responses in protection although this was largely dependent on the infectious parasite and the model used (primary or secondary infection, Ab-deficient) (Mukai et al., 2018). In some circumstances IgE-mediated activation of basophils also played a role (Karasuyama et al., 2018). Concerning the mediators implicated, these were rarely studied in these experiments although in many instances the production of cytokines appeared to play an important role (Mukai et al., 2018; Jimenez et al., 2021). Some studies also provided evidence for the role of proteases. Thus, Mcpt1 chymase-deficient mice had a markedly delayed worm expulsion and increased larval burden in *Trichinella spiralis* infection with the effect being majored after secondary infection (Knight et al., 2000). But, at the same time they showed less intestinal inflammation. Indeed, Mcpt1 can potentially degrade tight junction proteins such as occludin weakening the intestinal barrier thereby promoting intestinal inflammation (Knight et al., 2000; Lawrence et al., 2004). In a more chronic model, where the parasite had already infested skeletal muscles, it was rather Mcpt-6 tryptase that provided protection as live cysts increased in Mcpt-6-deficient mice due to a deficiency in anti-parasitic eosinophil infiltration (Shin et al., 2008). Proteases were also directly shown to kill parasites. Thus, SAG-1 surface antigen IgG-opsonized *Toxoplasma gondii* tachyzoites opsonized when co-cultured with MCs induced a polarized degranulation toward the parasite resulting in the tryptase-dependent parasite death (Joulia et al., 2015). While no specific role of histamine became apparent in helminth infection it appeared to play a detrimental role in malaria infection. Inhibition of histamine-mediated signaling conferred significant protection against severe malaria in mouse models of disease altering intestinal permeability (Beghdadi et al., 2008; Potts et al., 2016).

Concerning bacterial infection, the initial studies showing a protective effect of MC in bacterial sepsis indicated that it was the TNF cytokine prestored in MC LROs that conferred protection. Indeed, in this model TNF gets rapidly mobilized (<60 min) from LROs enabling rapid neutrophil infiltration, and the protective effect was abolished with an anti-TNF Ab (Echtenacher et al., 1996; Malaviya et al., 1996). As already mentioned above the lysosomal enzyme β-hexosaminidase released from LROs can inhibit bacterial growth and MC-deficient mice reconstituted with β-hexosaminidase-deficient MCs were more susceptible in a model of *Staphylococcus epidermidis* infection. It was shown that the enzyme degraded peptidoglycans of its bacterial cell wall, however, this microbicidal effect did not extend to *S. aureus* (Fukuishi et al., 2014). Proteases have also been implicated in anti-bacterial defense mechanism. Tryptase *Mcpt6* (−/−) mice were unable to eliminate *Klebsiella pneumoniae* from the peritoneal cavity with early extravasation of neutrophils to the peritoneal cavity being blunted (Thakurdas et al., 2007). In this context tryptase was shown to trigger the neutrophil attracting chemokine CXCL2 from endothelial cells. Mcpt4, the functional human chymase homologue was found to mediate protection in urinary tract infections caused by uropathogenic *E. coli* as it activated caspase-1 thereby triggering bladder epithelial cell death and shedding (Choi et al., 2016). In lower genital tract infections by group B *Streptococcus* (GBS) it was found to cleave fibronectin, reducing bacterial adherence (Gendrin et al., 2018). MCs can also produce and release upon degranulation the antimicrobial peptide cathelicidin (or LL37), which was shown to prevent invasive group A *Streptococcus* infection of the skin (Di et al., 2008).

Few studies have been performed analyzing the role of MC granular mediators in viral infection models. St. John and colleagues investigated the anti-dengue response. It is known that during secondary infection with a different serotype, poorly neutralizing Abs can enhance virus uptake leading to a cytokine storm, vascular permeability, and plasma leakage, which are hallmarks of MC activation. It was found that in such patients, disease severity was correlated with MC chymase and tryptase levels (Rathore and St, 2020). In an experimental model of dengue infection (Rathore and St, 2020; Syenina et al., 2015; Rathore et al., 2019), IgG-dependent MC activation induced vascular leakage in WT but not in MC-deficient mice. MC tryptase was identified as a key mediator by disrupting endothelial tight junctions (Rathore and St, 2020; Rathore et al., 2019).

So far, only a few studies have addressed the role of MCs in the immune response to fungi infections and even less is known concerning the released mediators (Jiao et al., 2019). The best studied model is *A. fumigatus* as it participates in allergic bronchopulmonary aspergillosis (ABPA) a severe allergic response further worsening existing lung diseases with detection of specific IgE antibodies (Urb et al., 2009). Although exposure to *Aspergillus fumigatus hyphae* leads to degranulation of MCs both in an IgE-independent and IgE-dependent manner, MCs do not seem to inhibit their growth or metabolic activity (Urb et al., 2009).

### 4.4 Role of LRO exocytosis in the circulatory system on immune responses

A study by Abraham’s group unexpectedly demonstrated that the dense cores of LROs, formed by electrostatic interactions between negatively charged heparin proteoglycans and positively charged insoluble mediators (e.g., TNF, MC-specific proteases), are secreted intact as submicrometric particles (Kunder et al., 2009). Strikingly, these secreted particles have demonstrated long-range functional activity by circulating through the lymphatic system to the draining lymph nodes, where they reinforcing immune responses (Kunder et al., 2009). As MC-derived particles are considered as a novel form of long-distance transport of inflammatory mediators, it has been proposed to use synthetic MC granules as adjuvants in vaccines to modulate immune responses (St John et al., 2012). MC-derived particles can pass through the spaces between endothelial cells into lymphatic vessels (Kunder et al., 2009). In blood vessels, studies demonstrate that perivascular MCs interact directly with blood vessels by extending cytoplasmic projections into the vascular lumen (Dudeck et al., 2021; Link et al., 2024; Cheng et al., 2013). Upon activation (e.g., exposure to DNFB, IgE cross-linking), these MCs release LRO contents directionally into the bloodstream. This polarized degranulation ensures the diffusion of mediators such as TNF into the circulation, enabling the recruitment of neutrophils at the inflammatory site (Dudeck et al., 2021). The close contacts between MCs and vascular endothelium is dependent on integrin β1. Indeed, genetic deletion of *Itgb1* gene in MCs disrupted these interactions and abrogated anaphylactic responses to blood-borne allergens, highlighting its critical role in MC-mediated vascular communication (Link et al., 2024).

## 5 Conclusive remarks

Unwittingly visualized almost two centuries ago, MC LROs play a pivotal role in the immune surveillance function of MCs. These organelles exhibit dual functionality, mediating protective effects such as microbial defense and venom detoxification, while also contributing to pathological outcomes like allergic inflammation, depending on the context of MC activation. Research on LROs has considerably enriched our understanding of the molecular mechanisms that govern their biogenesis, transport, secretion and overall function, notably through the use of genetically engineered mouse models. Future studies will focus on novel protocols combining functional genomics (based on RNA interference or CRISPR-Cas9 genome editing) with high-resolution confocal microscopy enabling the precise identification of new regulators of MC degranulation (Folkerts et al., 2020). The studies of LRO composition have identified histamine as a key mediator of allergic reactions, leading to the development of anti-histamines as therapeutic agents. Additionally, insights into the inherent properties of their dense core, capable of long-distance transport to lymph nodes via the bloodstream, have enabled the development of synthetic MC granules. These engineered particles have been explored as tools to modulate immune responses in vaccine development. Thus, in the future, targeting specific genes expressed in MCs that regulate LRO maturation and secretory pathways may enable the development of novel therapeutics treatments for both allergic and non-allergic diseases driven by MCs.
